# Fatigue characteristics and mechanism of phosphogypsum stabilised red clay under dry and wet cycles

**DOI:** 10.1371/journal.pone.0331317

**Published:** 2025-09-05

**Authors:** Yan Hu, Kaisheng Chen, Yuang Chen, Jinxiong Chen, Kai Zhang

**Affiliations:** 1 School of Civil Engineering, Guizhou University, Guiyang, Guizhou Province, China; 2 School of Civil Engineering, Southwest Jiaotong University, Sichuan Province, China; University of Sharjah, UNITED ARAB EMIRATES

## Abstract

Phosphogypsum is an acidic solid waste mainly composed of CaSO₄-2H₂O by-products of the wet process phosphoric acid industry, which has the characteristics of high impurity content, poor stability of stockpiling, but can be utilized in a resourceful way. Phosphogypsum waste utilization can reduce environmental pollution, save resources and create economic value. In order to investigate the fatigue characteristics and the mechanism of dynamic strength change of cement-phosphogypsum-red clay under wet and dry cycles, the cumulative deformation characteristics and the rule of change of critical dynamic stress of the mixed materials were investigated by dynamic triaxial fatigue test, SEM and XRD test, and the mechanism of dynamic strength change was analyzed according to the microstructure and the chemical mineral composition of the mixed materials. The test results show that: the cumulative deformation curve of the mix under the action of dry and wet cycles is divided into three types: stable, destructive and critical, and the critical dynamic stress is positively related to the peripheral pressure and consolidation ratio, and negatively related to the number of dry and wet cycles; the mechanism of dynamic strength change: the hydration of cement generates hydrated calcium silicate gel, which coalesces fine particles of the mix to form agglomeration, and the calcium sulfate dihydrate in phosphogypsum reacts with it to generate calcium alumina, and at the same time, the particles form clusters through electrostatic adsorption, and the particles of calcium sulfate dihydrate react with it to form calcium alumina. Through electrostatic adsorption to form clusters, a variety of agglomerates intertwined to form a stable structure with a certain strength. Relative to other proportions of phosphogypsum and red clay, the mix has better dynamic strength at a 1:1 mass ratio of phosphogypsum to red clay; and too much phosphogypsum will make the mix acidic increase, resulting in some of the calcium alumina dissolved, while the dry and wet cycle will increase the internal pores of the mix, reducing the strength of the mixture.

## 1. Introduction

Red clay is a clay rich in iron and aluminum oxides, widely distributed in tropical and subtropical regions such as southern China, Southeast Asia, Africa and South America. Red clay is often used as roadbed fill in construction, but due to its high liquid limit, high pore ratio and significant water sensitivity, it is susceptible to water softening and water loss and cracking in its natural state, leading to settlement deformation and slope instability [1–3]. In order to improve its engineering performance, it is usually necessary to mix cement, lime or industrial waste residue and other curing agents, or can be inhibited by additives to inhibit expansion [4–7]. Strict control of water content, enhanced drainage measures, and avoidance of rainy season operations are required during construction to overcome its limitations such as poor permeability and compaction difficulties. Phosphogypsum is a by-product of industrial wet preparation of phosphate fertilizers. It has been studied that for every 1t of phosphoric acid generated, 5t of phosphogypsum is produced as a by-product.[8] The global utilization rate of phosphogypsum is low, averaging about 15–20%.China is the country that produces the largest amount of phosphogypsum, with an annual production of more than 100 million tons, but the utilization rate is only about 40%.At present, phosphogypsum is mainly disposed of by means of surface landfills and on-site stockpiling. Since it contains a small amount of phosphorus, fluorine and other impurities, these treatments cause pollution to the environment and at the same time occupy a large amount of land resources, so its resource utilization is of great significance. Current studies have shown that phosphogypsum can be comprehensively utilized for road construction materials, mine filling, cement retardant, etc [9–10].

Roadbed stability plays an important role in the construction and use of roads. During vehicle traffic, cyclic dynamic loads are continuously generated and applied to the roadbed, which in the long term will lead to cumulative deformation of the roadbed, thus affecting the dynamic stability of the roadbed [11]. Cai [12] and others used cement modified expansive soil as the research object and pointed out that the dynamic parameters of the roadbed in the impregnated state are larger than the natural state values, and the roadbed impregnation will promote the cumulative deformation of the roadbed. Li Jinhong [13] explored the dynamic response characteristics of low red clay embankment under vehicle loading by modeling tests and numerical simulations, The results show that the vertical dynamic stresses and accelerations decrease rapidly and then slowly along the depth and lateral width directions; Increased vehicle loads and speeds result in greater vertical dynamic stresses and accelerations, while higher modulus of elasticity and embankment thickness reduce these responses. Increased moisture content exacerbates the vertical acceleration response. Feng Xumao et al [14] found that water immersion in the roadbed significantly weakened the resistance of the roadbed to dynamic stresses, leading to an increase in the dynamic response and cumulative deformation through driving tests and excitation tests. Long Ningbo et al [15] used cement and basalt fibers to improve red clays. The dynamic strength and maximum dynamic modulus of elasticity of the modified soil increased significantly, the damping ratio decreased significantly, and the form of its dynamic damage was changed. Wei-Qiang Cao [16] et al. used coir fibers to reinforce red clay and found that coir fibers can increase the strength and ductility of red clay through unconfined compressive strength test and triaxial test. Huang Wendong et al [17] investigated the variation rules of dynamic strain, damping ratio and dynamic shear modulus of cement-phosphogypsum-red clay by dynamic triaxial tests. Huang Tao et al [18] found that the waterproofed phosphogypsum cementitious materials have good volume stability. Zhang [19] concluded that the water resistance of phosphogypsum cementitious materials is better than that of ordinary gypsum cementitious materials, moreover, the mass loss of cementitious materials increases with increasing temperature. Lei [20] analyzed the effects of freezing and thawing cycle frequency, freezing temperature and perimeter pressure on the cumulative plastic strain of roadbed soil, and established an empirical model to predict the cumulative plastic strain of soil.

From the above, it can be seen that although the existing studies have deeply explored the engineering properties of roadbed soil in different natural states, they have generally neglected the influence of complex climatic conditions such as dry and wet cycles on the long-term performance of highways, and the roadbed soil is inevitably subject to dry and wet cycles in the actual project, in view of this situation, this study adopts dynamic triaxial fatigue test to explore the dynamic properties of cement-phosphogypsum-red clay under dry and wet cycles In this study, the dynamic triaxial fatigue test was used to investigate the change rule of cement-phosphogypsum-red clay dynamic properties under wet and dry cycles, and the change mechanism of dynamic strength was revealed from microstructure and chemical composition. The research results provide a scientific basis for the application of phosphogypsum in highway engineering, and also provide a reference for improving the engineering properties of red clay, which is of great scientific significance and engineering application value.

## 2. Test materials and programs

### 2.1. Test raw materials

The red clay used in the test was taken from Huaxi District, Guiyang City, with the depth of 0–3 m. The surface of the soil samples showed reddish-brown color, loose texture, high natural water content, strong cohesion, and belonged to the high-liquid-limit clay. Its basic physical parameters are shown in Table 1.

**Table 1:** Basic physical indexes of red clay.

Natural density/g‧cm^-3^ 1.765	Natural moisture content/%	Unevenness coefficient	Curvature factor	Liquid limit/%
	49.49	8.60	1.40	80.40
Plastic limit/%	Plasticity index	Optimum moisture content/%	Maximum dry density/g‧cm^-3^	
49.60	30.80	23.74	1.452	

The phosphogypsum was taken from the urnfu phosphorus mine in Fuquan City, Guizhou Province, with a grayish-white appearance. The basic physical indexes, chemical composition, and heavy metal and radioactivity detection results of phosphogypsum are shown in Tables 2–3. According to the heavy metal and radioactivity test results, it can be seen that the heavy metal content and radioactivity index of the phosphogypsum used in the test are in line with the relevant provisions of the national standard (GB 5085.3−2007) respectively.

**Table 2:** Basic Parameters of Phosphogypsum and Phosphogypsum chemical composition.

Basic parameters of phosphogypsum	Specific surface area/ m^2^‧kg^-1^	102
Heat loss/%	18.43
Moisture content/%	5.3
Densities/g‧cm^-3^	2.38
Fineness/%	44.3
Phosphogypsum chemical composition mass fraction (%)	SO_3_	49.070
	CaO	40.070
	SiO_2_	5.780
	P_2_O_5_	1.350
	Na_2_O	0.587
	Al_2_O_3_	0.435
	else	2.708

**Table 3:** Test Results of Heavy Metals and Radioactivity in Phosphogypsum.

Sports event		Limit value	Test results	Reach a verdict
Heavy metal element	Cu/mg‧L^-1^	≤100	0.157	eligible
	Zn/mg‧L^-1^	≤100	0.051	eligible
	Cd/mg‧L^-1^	≤1	0	eligible
	Pb/mg‧L^-1^	≤5	0	eligible
	Cr/mg‧L^-1^	≤15	0	eligible
	As/mg‧L^-1^	≤5	0.0356	eligible
	Hg/mg‧L^-1^	≤0.1	0.0005	eligible
Radiant	Ra-226/Bq‧kg^-1^	—	53.94	—
	Th-232/Bq‧kg^-1^	—	42.13	—
	K-40/Bq‧kg^-1^	—	52.95	—
	Internal irradiance index (IRI)	≤1.0	0.3	eligible
	External irradiance index (EI)	≤1.0	0.3	eligible

The cement was P.C32.5R silicate cement with dark gray appearance and uniform particles, and its basic indexes are shown in Table 4.

**Table 4:** Basic parameters of cement.

Heat loss/%	SO3/%	MgO/%	Specific surface area/m^2^‧kg^-1^	Incipient condensation time/min	Time of final coagulation/min
4.14	2.20	1.98	348	166	221
Stability	Chloride ion/%	Gypsum admixture/%	Grinding aids/%	3-Day Flexural Strength/MPa	3-day compressive strength/MPa
eligible	0.018	5.00	0.1	5.9	29.2

### 2.2. Pilot program

#### 2.2.1. Sample preparation.

Peng Bo [21] investigated the strength characteristics of cemented phosphogypsum stabilized soil through indoor tests and found that the mechanical properties and economy of the mixture were optimal when the cement dosing was 4% to 6% and when cement: phosphogypsum = 1:2. Zhou Mingkai [22] et al. found that when cement phosphogypsum stabilized crushed stone was tested for unconfined compressive strength, it was found that the unconfined compressive strength reached the maximum value when the cement dosage was 5%. Liu Chao et al [23], when studying the performance of cement-phosphogypsum stabilized crushed stone pavement base material, pointed out that at 8% phosphogypsum doping, the 7 and 28 d strength of the material reached the optimum value. Also according to Table 4.6.4 of the Technical Rules for the Construction of Highway Pavement Subgrade (JTG/T F20-2015) [24], when the stabilized soil is used as a sub-base and the plasticity index is > 12, the recommended cement dosage is 6% to 14%. Excessive amount of cement makes the mixture easy to crack. Combining various factors, the cement dosage is proposed to be 4%, 6% and 8%. On this basis, this study further investigates the dynamic stability of mixed materials with different ratios subjected to long-term dynamic loading under dry and wet cycles. A total of 18 ratio mixes in six groups of phosphogypsum: red clay = 2:1, 1:1, 1:2, 1:3, 1:4 and 1:5 were tested. Highway Roadbed Design Specification (JTG D30-2015) [25] requires that the compaction degree of roadbed fill is not less than 90% for three- and four-level highways, and 96% for one-level highways, and the compaction degree is 96% for this test. Studies have shown that when red clay is used as roadbed fill, the optimum water content can ensure the strength and stability of the roadbed soil [26–27], thus the water content of the specimen is taken as the optimum water content. The maximum dry density and optimum moisture content of different mix ratios were obtained by heavy-duty compaction test, the specific results are shown in Table 5. When making samples, firstly mix the dried phosphogypsum with red clay, add 90% of the required water and mix evenly, after 24h of curing, add the corresponding amount of cement into the mixture, add the remaining 10% of water and mix evenly, and then make samples of the finished mixture by static pressure method. The specimen size was Φ39.1 × 80 mm, and the sample making process must be completed within 1h to prevent the cement from curing. The prepared specimens were placed in a curing box with a relative humidity of more than 95% and a temperature of 20 ± 2°C for 7d.

**Table 5:** Optimum moisture content and maximum dry density of mixes with different proportions.

P:T	4% C		6% C		8% C	
	Optimum moisture content/%	Maximum dry density/g‧cm-3	Optimum moisture content/%	Maximum dry density/g‧cm-3	Optimum moisture content/%	Maximum dry density/g‧cm-3
2:1	20.30	1.53	19.40	1.52	18.90	1.56
1:1	21.30	1.56	21.80	1.56	20.80	1.57
1:2	22.50	1.54	23.10	1.55	21.80	1.56
1:3	23.46	1.52	24.10	1.54	22.90	1.54
1:4	24.36	1.45	25.60	1.51	24.50	1.50
1:5	26.00	1.46	26.70	1.50	25.70	1.50

Note: In this paper, C stands for cement, P stands for phosphogypsum, and T stands for red clay.

#### 2.2.2. Pilot program.

The prepared and maintained specimens were first subjected to wet and dry cycles.The specimens after wet and dry cycles were subjected to dynamic triaxial fatigue test and micro-mechanism test respectively.

(1) Wet/dry cycle method

According to the research results of Xie Huihui [28] and Li Zhen et al [29], the degree of attenuation of the unconfined compressive strength of red clay by first wetting and then drying is much greater than that of first drying and then wetting. Hu Zhi et al [30] showed that: the roadbed was compacted and filled at the optimum moisture content, and after a period of time in the natural climatic environment, the moisture content of the roadbed soil increased and fluctuated cyclically or non-cyclically within the range of 5% above and below the equilibrium moisture content (EMC); and Dong Wei et al [31] showed that: the performance of the roadbed soil usually stabilized after 4 ~ 6 dry and wet cycles. and continued increase in the number of cycles had a limited effect on the characterization of the performance evolution. Xu Gangmin [32] found that the unconfined compressive strength of mixes with different phosphogypsum dosage decreased with the increase of the number of wet and dry cycles. Among them, the fastest decrease was observed in the first three times, and then gradually leveled off. In summary, the wet and dry cycle wet and dry path is first selected as first wet and then dry, the number of wet and dry cycle is formulated as 5 times, the wet and dry moisture content up and down amplitude is taken as 10%, that is, the target moisture content in the humidification stage is the optimal moisture content +5%, and the target moisture content in the drying stage is the optimal moisture content −5%, as shown in Table 6.

**Table 6:** Dry and wet cycle moisture content.

P:T	4% C			6% C			8% C		
	Optimum moisture content/%	Humidified moisture content/%	Dry moisture content/%	Optimum moisture content/%	Humidified moisture content/%	Dry moisture content/%	Optimum moisture content/%	Humidified moisture content/%	Dry moisture content/%
2:1	20.30	25.30	15.30	19.40	24.40	14.40	18.90	23.90	13.90
1:1	21.30	26.30	16.30	21.80	26.80	16.80	20.80	25.80	15.80
1:2	22.50	27.50	17.50	23.10	28.10	18.10	21.80	26.80	16.80
1:3	23.46	28.46	18.46	24.10	29.10	19.10	22.90	27.90	17.90
1:4	24.36	29.36	19.36	25.60	30.60	20.60	24.50	29.50	19.50
1:5	26.00	31.00	21.00	26.70	31.70	21.70	25.70	30.70	20.70

**Fig 1:**
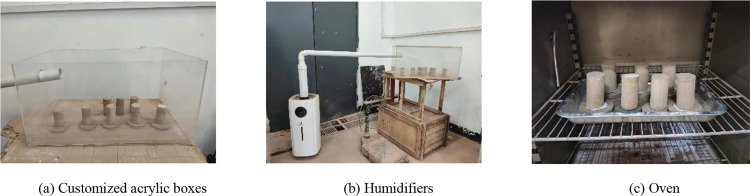
Dry and wet cycle experimental apparatus.

**Fig 2:**
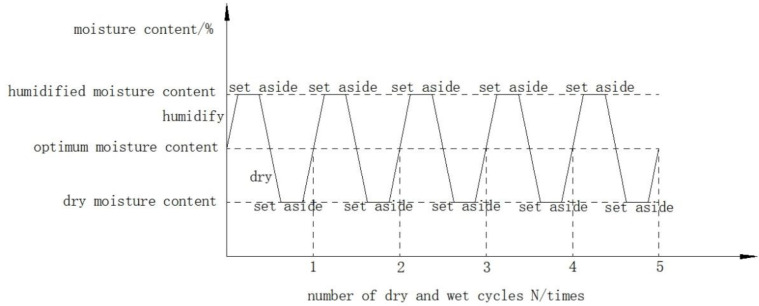
Schematic diagram of wet and dry cycle process.

The instruments used in the dry and wet cycle test are customized acrylic box (L × W × H = 60 cm × 40 cm × 30 cm), humidifier (maximum humidification speed 2000ml/h, rated capacity 21L), PVC pipes, pads, permeable stones, oven, electronic scales, etc. The main test instruments are shown in Fig 1, and the wet and dry process is shown in Fig 2 [33]. Humidification first in the bottom of the acrylic box placed in the permeable stone, the standard maintenance of 7d specimens placed on the permeable stone, to the box of water, the height of water injection should not exceed the thickness of the permeable stone, and then the top of the acrylic box with a humidifier humidifier, every 1h determination of the moisture content of the specimen, when the water content reaches the target rate to stop the humidification and static specimens for 24h to make the moisture uniformity; static drying with a 40°C oven, timed measurement The moisture content, when the moisture content reaches the target moisture content of drying, stop drying, static specimen 24h, continue to humidify to the optimal moisture content, so that a complete wet and dry cycle, repeat the above process to get different wet and dry cycle times of the specimen.

(2) Dynamic triaxial fatigue test program

**Fig 3:**
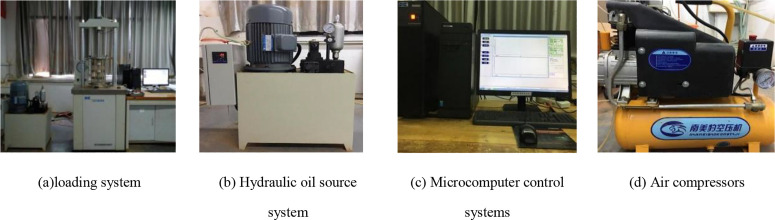
SDT-20 Dynamic Triaxial Tester.

Fig 3 shows the SDT-20 dynamic triaxial testing machine for triaxial fatigue test. The apparatus mainly consists of a loading system (including a peripheral pressure application system, an axial loading mechanism and frame, and a pressure chamber), a hydraulic oil source system, a gas and water application piping system, a microcomputer control section, an air compressor, an electrical control section, and a data acquisition and analysis system. The system provides axial dynamic load upper limit value of 20kN, range of 2 cm, measuring strain accuracy of 10−4, load average fluctuation and deformation accuracy are higher than 0.05, the maximum peripheral pressure of the pressure chamber is 1MPa, and it can simulate trapezoidal, square, sinusoidal and triangular waves.

The dynamic load on the roadbed generated by the train movement is dominated by low frequency, so the vibration frequency is taken as 5.0 Hz and the consolidation ratios are taken as 1, 1.5 and 2. The perimeter pressure of the roadbed filler is generally 30 ~ 80kPa, the test perimeter pressure is taken as 40kPa, 80kPa, 120kPa, and the number of dry and wet cycles is 0 ~ 5 times. Yu Zhou [34] showed that when the number of vibration is 104 times, the residual deformation of saturated soil tends to stabilize, so the maximum number of vibration of the test is taken as 104 times, the dynamic triaxial fatigue test program is shown in Table 7. Take C:P:T = 6:47:47 as an example, its dynamic stress amplitude is shown in Table 8, when the first level of dynamic stress amplitude test is over, to 20kPa for the increase in dynamic stress step by step, each level of vibration 104 times after entering the next level. Cumulative deformations are automatically collected by SDT-20 computerized data collector.

**Table 7:** Dynamic triaxial fatigue test programme.

Frequency/(Hz)	Pressurization (kPa)	Consolidation ratio	Number of cycles*N*/times
5	40	1	0、1、2、3、4、5
		1.5	0、1、2、3、4、5
		2	0、1、2、3、4、5
5	80	1	0、1、2、3、4、5
		1.5	0、1、2、3、4、5
		2	0、1、2、3、4、5
5	120	1	0、1、2、3、4、5
		1.5	0、1、2、3、4、5
		2	0、1、2、3、4、5

**Table 8:** Dynamic stress amplitude values for the mix ratio 6:47:47.

Consolidation ratio	Pressurization/kPa	Dry and wet cycle number of cycles/times	Initial dynamic stress amplitude/kPa	Dynamic stress amplification/kPa
1	40	0 ~ 5	160,140,130,120,100,80	20
	80		200,180,160,140,130,120	20
	120		220,200,180,160,150,140	20
1.5	40	0 ~ 5	220,200,160,140,130,120	20
	80		260,220,180,160,150,140	20
	120		280,230,200,180,160,150	20
2	40	0 ~ 5	240,220,180,160,150,140	20
	80		280,240,200,180,160,150	20
	120		300,280,260,240,220,180	20

(3) SEM and XRD tests

Microscopic testing of the specimens was accomplished by SEM scanning electron microscopy and XRD compositional characterization. Scanning electron microscope model HITACHI SU8100 with accelerating voltage of 3.00 kV. The sample was broken under different ratios and number of wet and dry cycles. The middle portion of the soil block was taken and polished with sandpaper to a flake of about 1 cm^3^.The residual powder on the surface of the sample was cleaned up to make a clean sample surface. The mixture was required to be sprayed with a gold coating prior to scanning because of its poor electrical conductivity. X-ray diffractometer model Rigaku SmartLab, scanning step size of 0.02 °, scanning range of 15 ° 80 °, scanning rate of 10 (°)/ min, the specimen is taken from the center of the sample of the soil, crushed to powder for compositional analysis, the test program is shown in Table 9.

**Table 9:** Table of SEM and XRD test protocols.

Proportion/P:T	Cement admixture/%	Moisture content/%	Compaction/%	Number of wet and dry cycles
2:1 1:1	6	21.80 (optimal) 23.10 (optimal)	96	0、5 0、5

## 3. Analysis of test results

The cumulative deformation curves of materials are categorized into 3 types: stable, critical and destructive. At the beginning of the cyclic dynamic load, the cumulative deformation with the increase in the number of vibration and slowly increase, the filler in the vibration under the action of the filler becomes dense, at this time the level of dynamic stress is low, when the number of vibration reaches a certain value of the cumulative deformation of the material tends to stabilize, the curve is called the stabilizing type curve. At higher dynamic stress levels, the cumulative deformation increases with the number of vibrations until the roadbed is destroyed, which is called a destructive curve. When the cumulative deformation with the increase in the number of vibration is large and small, fluctuating up and down in a certain interval, the curve is between the stable and destructive, the curve is called the critical type curve. Liu Xiaohong [35] used (1) to describe the stabilizing type curve and Cai Ying [36] used equation (2) to describe the destructive type curve. For the critical type curve, due to the special characteristics of the curve itself, no scholars have proposed a mathematical model with a better fitting effect, and (3) is used to describe it.


$$\begin{array}{c} \varepsilon_{p}=\frac{\alpha *N^{\beta }}{1+\gamma *N^{\beta }} \end{array}$$ (1)

$$\begin{array}{c} \varepsilon_{p}=AN^{B} \end{array}$$ (2)

$$\begin{array}{c} \varepsilon_{p}=a+bN \end{array}$$ (3)

$\varepsilon_{p}$ is the cumulative plastic strain, N is the number of loadings, a, b, α, β, and γ are fitting parameters related to the dynamic stress level, soil properties, and other factors.

The actual dynamic stress is less than a certain value of σ_dc_ with the increase in the number of vibration cumulative deformation tends to stabilize, on the contrary, when the actual dynamic stress is greater than the fixed value of σdc, with the increase in the number of vibration cumulative deformation continues to increase until the material is destroyed, at this time the σ_dc_ is called the critical dynamic stress. The critical dynamic stress is theoretically a fixed value, but due to the limitation of test conditions, the critical dynamic stress can only be obtained as a range and its specific value cannot be derived.

### 3.1. Cumulative deformation of the mix

**Fig 4:**
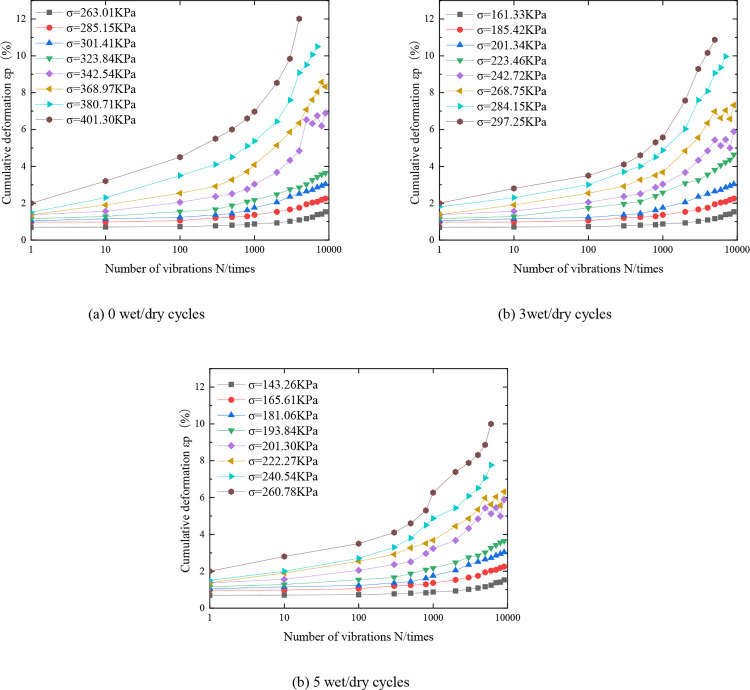
Cumulative deformation εp ~ N curves of mixes with different number of wet and dry cycles.

The variation of cumulative deformation of the mix with the number of vibrations for different numbers of wet and dry cycles is shown in Fig 4. Limited to space, only show the case of C:P:T = 6:47:47, peripheral pressure of 80kPa, consolidation ratio of 1.5, vibration frequency of 5 Hz, and the number of dry and wet cycles of 0, 3, and 5. There are similar laws for other cases, which will not be repeated. From the characteristics of the cumulative deformation curve of the mixture, it can be seen that the cumulative deformation curve belongs to one of the three types of stable, destructive and critical, the cumulative deformation of the mixture is positively correlated with the number of vibration and the level of dynamic stress, and the greater the dynamic stress, the faster the growth rate of the cumulative deformation, therefore, reduce the dynamic stress or reduce the number of vibration can effectively improve the dynamic stability of the roadbed.

Taking 3 times of wet and dry cycles as an example, it can be seen from Fig 4(b) that when the dynamic stress is 161.33kPa (the cyclic load in the test is fluctuating, so there is a slight difference between the actual load and the set value, which is 160kPa, respectively), and the number of vibration times is 0 ~ 700, the cumulative deformation curve is close to a horizontal straight line, and the specimen is almost not deformed.

When the number of vibration is 700 ~ 1000, the cumulative deformation curve is similar to a slanting straight line, at this time the specimen is mainly elastic deformation. When the number of vibration is greater than 1000, the cumulative deformation of the specimen gradually increases, and the slope of the curve is improved, the specimen is mainly elastic deformation, accompanied by a small amount of plastic deformation, the cumulative deformation curve under the dynamic stress is relatively smooth, in line with the characteristics of the stabilized curve. The dynamic stresses of 185.42 kPa, 201.34 kPa, and 223.46 kPa have similar patterns, and by the same token, it can be seen that the cumulative deformation curves under these three levels of dynamic stresses are all stable.

Dynamic stress is 242.72 kPa, the number of vibration is greater than 6000, the cumulative deformation curve with the increase in the number of vibration up and down fluctuations, at this time the specimen elastic deformation and plastic deformation, and has not yet been destabilized, according to which the curve belongs to the critical type, and similarly know that when the dynamic stress is 268.75 kPa is also belongs to the critical type curve. When the dynamic stress is 284.15 kPa and the number of vibration is more than 1000 times, the deformation of the specimen expands rapidly, a large amount of plastic deformation is produced, and the specimen is destroyed, the curve is destructive, and the cumulative deformation curve when the dynamic stress is 297.15 kPa is also destructive.

According to the characteristics of the cumulative deformation curves in Fig 4, the dynamic triaxial fatigue test data were fitted with Eqs. (1), (2), and (3), and the fitting results are shown in Tables 10–12. From the fitting results, it can be seen that the stabilized type in the number of dry and wet cycles 0, 3 times R^2^ value are greater than 0.9, when the number of dry and wet cycles to 5 times, R 2 value slightly reduced but the minimum value is still greater than 0.8, the fitting effect is good. Critical type dry and wet cycle times 0, 3 times R 2 values are less than 0.9, when the number of dry and wet cycle times reached 5 times, R^2^ values decreased to 0.7, the fitting effect is general. The R 2 value of the destructive type is greater than 0.95 when the number of dry and wet cycles is 0, 3 and 5 times, and the fitting effect is good. The good fitting effect of stabilizing type and destructive type indicates that Eqs. (1) and (2) can better describe the relationship between the cumulative deformation and vibration times of cement-phosphogypsum-red clay under low peritectic pressure and the number of dry and wet cycles; the fitting effect of the critical type curve is average.

**Table 10:** Cumulative deformation curve fitting parameters for 0 wet and dry cycles of mixes.

Curve type	Dynamic stress amplitude setting/kPa	Dynamic stress amplitude measured value/kPa	α,a,A	β,b,B	γ	R^2^
stabilized	260	263.01	0.03169	0.43361	0.01480	0.91094
stabilized	280	285.15	0.00701	0.91446	0.00563	0.96323
stabilized	300	301.41	0.14352	0.43149	0.03352	0.96281
stabilized	320	323.84	0.09815	0.59101	0.02350	0.96453
critical	340	342.54	1.94364	0.00044	---	0.84728
critical	360	368.97	2.41525	0.00067	---	0.83705
destructive	380	380.71	0.35797	0.43580	---	0.98230
destructive	400	401.3	0.47937	0.46391	---	0.95675

**Table 11:** Cumulative deformation curve fitting parameters for 3 wet and dry cycles of mixes.

Curve type	Dynamic stress amplitude setting/kPa	Dynamic stress amplitude measured value/kPa	α,a,A	β,b,B	γ	R^2^
stabilized	160	161.33	0.00370	0.73071	0.00473	0.94258
stabilized	180	185.42	0.00522	1.00106	0.00360	0.90689
stabilized	200	201.34	0.09470	0.46181	0.02885	0.95492
stabilized	220	223.46	0.25391	0.35622	0.02550	0.98007
critical	240	242.72	1.87610	0.00045	---	0.85771
critical	260	268.75	2.27895	0.00066	---	0.86605
destructive	280	284.12	0.40704	0.39665	---	0.99039
destructive	300	297.25	0.44113	0.43261	---	0.98805

**Table 12:** Cumulative deformation curve fitting parameters for 5 wet and dry cycles of mixes.

Curve type	Dynamic Stress amplitude setting/kPa	Dynamic stress amplitude measured value/kPa	α,a,A	β,b,B	γ	R^2^
stabilized	140	143.26	0.07365	0.23744	0.11066	0.84888
stabilized	160	165.61	0.02409	0.79659	0.01924	0.89569
stabilized	180	181.06	0.08023	0.70132	0.03574	0.92444
stabilized	190	193.84	0.09464	0.55516	0.02136	0.96382
critical	200	201.30	2.25006	0.00040	---	0.73010
critical	220	222.27	2.41597	0.00061	---	0.88115
destructive	240	240.54	0.81885	0.28199	---	0.99501
destructive	260	260.78	1.09132	0.27924	---	0.99662

### 3.2. Critical dynamic stresses in mixes

#### 3.2.1. Effect of the number of wet and dry cycles.

**Fig 5:**
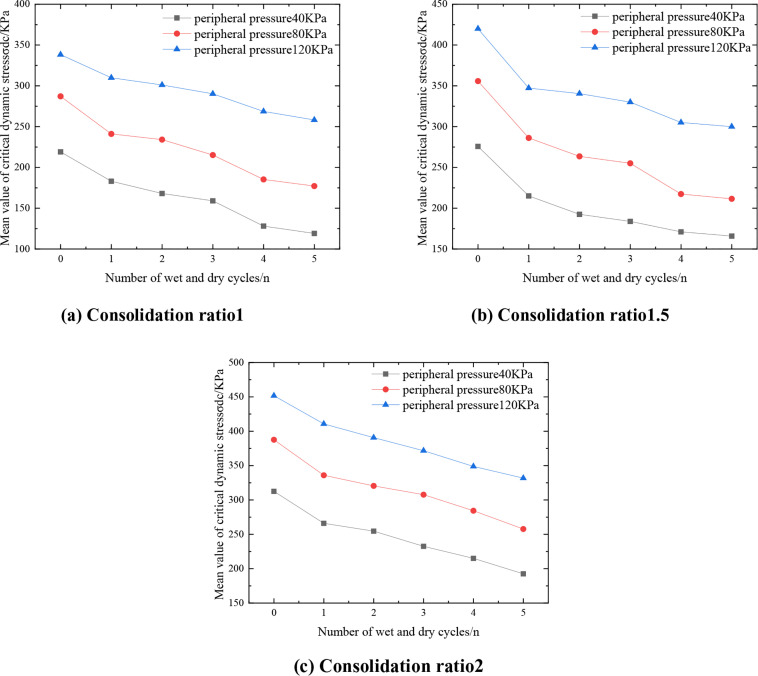
Curve of critical dynamic stress versus the number of wet and dry cycles.

The mean value within the critical dynamic stress range is taken as the representative value of the critical dynamic stress. C:P:T = 6:47:47, and the relationship between the representative values of critical dynamic stresses in the mix and the number of wet and dry cycles is shown in Fig 5. As can be seen in Fig 5, the critical dynamic stress is negatively correlated with the number of wet and dry cycles, and the representative value of the critical dynamic stress decreases the most after the first wet and dry cycles, indicating that wet and dry cycles will reduce the ability of the mix to resist dynamic instability, which is due to the process of humidification, hydrophilic material inside the mix absorbs water leading to the expansion of the specimen, and the drying process of the specimen is not uniformly heated inside the oven and the specimen loses water to shrinkage and deformation, and the temperature gradient and shrinkage and deformation together produce tensile stress inside the specimen. Under the joint action of temperature gradient and shrinkage deformation, tensile stress is generated inside the specimen, when the tensile stress is greater than the mix’s own tensile strength, then cracks will be generated, with the increase in the number of wet and dry cycles, the cracks are further developed, thus reducing the critical dynamic stress of the mix. Taking the peripheral pressure of 40kPa and consolidation ratio of 1.5 as an example, the representative value of critical dynamic stress of the mixture without wet and dry cycles is 275.75kPa, and after 1 wet and dry cycle, its critical dynamic stress decreases to 215kPa, which is a decrease of 22.03%; after 2 wet and dry cycles, the representative value of critical dynamic stress of the mixture is 192.43kPa, which is a decrease of 10.49%. This law has an important guiding value for the life prediction of roadbed engineering in hot and humid areas, which can be extended by reserving sufficient strength redundancy in the design or by adopting protective measures such as surface closure to block moisture exchange.

#### 3.2.2. Impact of peripheral pressure.

**Fig 6:**
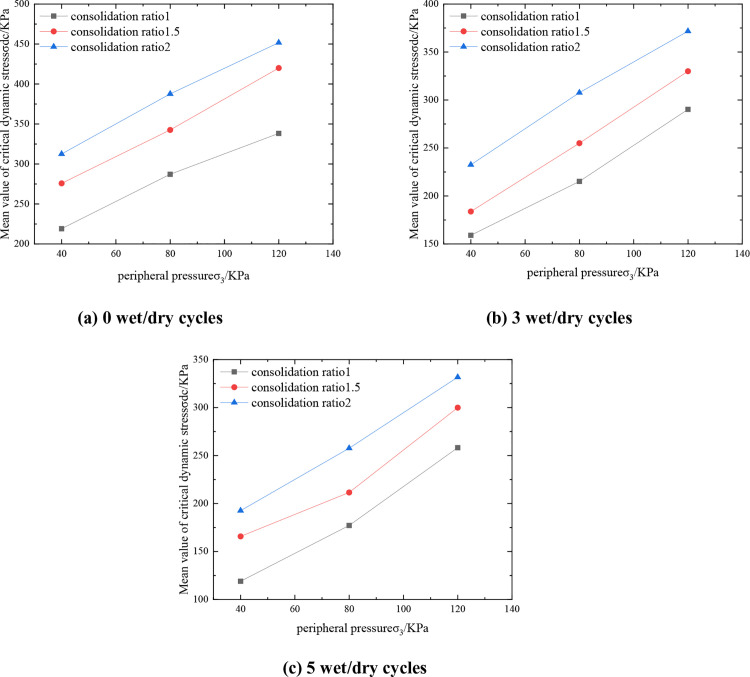
Variation curve of mean critical dynamic stress-consolidation ratio of mixture.

Fig 6 shows the relationship between C:P:T = 6:47:47, the representative value of the critical dynamic stress of the mixture and the surrounding pressure. From Fig 6, it can be seen that under the same consolidation ratio, the representative value of the critical dynamic stress of the mixture is positively correlated with the perimeter pressure and close to a linear relationship, which is due to the fact that when the perimeter pressure is increased, the extrusion makes the spacing of particles inside the mixture become smaller to form a tighter skeleton structure, the material is more compact, and the friction between the soil bodies increases, thus increasing its critical dynamic stress. Taking three dry and wet cycles with consolidation ratio of 1.5 as an example, when the perimeter pressure was increased from 40kPa to 80kPa, the representative value of critical dynamic stress of the mix changed from 183.7 to 255kPa, which was an increase of 38.8%. Enough compaction should be ensured in the roadbed construction to improve the initial perimeter pressure; pre-pressure method can be used or lateral restraint structure can be set up for soft ground; in the design of dynamic load area, the long-term dynamic stability of the soil body should be reasonably assessed according to the actual state of perimeter pressure.

#### 3.2.3. Effect of consolidation ratio.

**Fig 7:**
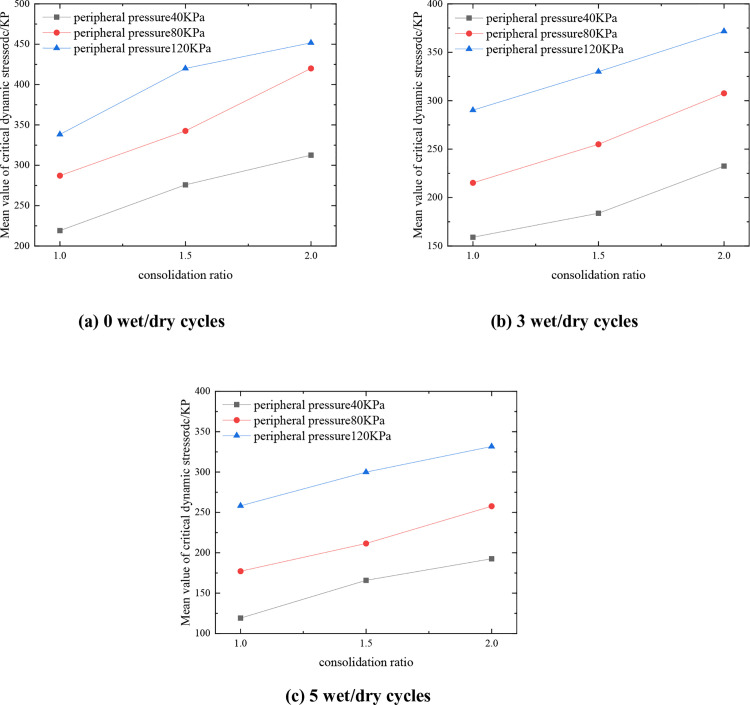
Variation curve of mean critical dynamic stress-consolidation ratio of mixture.

Fig 7 shows the curves of the critical dynamic stress representation of the mix with respect to the consolidation ratio for C:P:T = 6:47:47. From Fig 7, it can be seen that under the same circumferential pressure, with the increase of the consolidation ratio, the contact between the soil body is more compact, forming a more stable skeleton structure, which effectively improves the overall resistance to deformation of the mixture, and the representative value of the critical dynamic stress of the mixture increases. Taking 0 wet and dry cycles as an example, the critical dynamic stress representative value of the mix is 287.12 kPa when the perimeter pressure is 80 kPa and the consolidation ratio is 1. When the consolidation ratio is increased to 1.5, the critical dynamic stress representative value is 342.54 kPa, which is an increase of 55.42 kPa or 19.30%. This law has important guiding significance for engineering practice: in roadbed filling and foundation treatment, pre-compaction or layered rolling process can be used to improve the actual consolidation ratio and enhance the long-term dynamic stability of the structure; at the same time, in the laboratory ratio design, the stress state of the actual project should be simulated to determine the optimal consolidation parameters.

#### 3.2.4. Influence of fit ratio.

**Fig 8:**
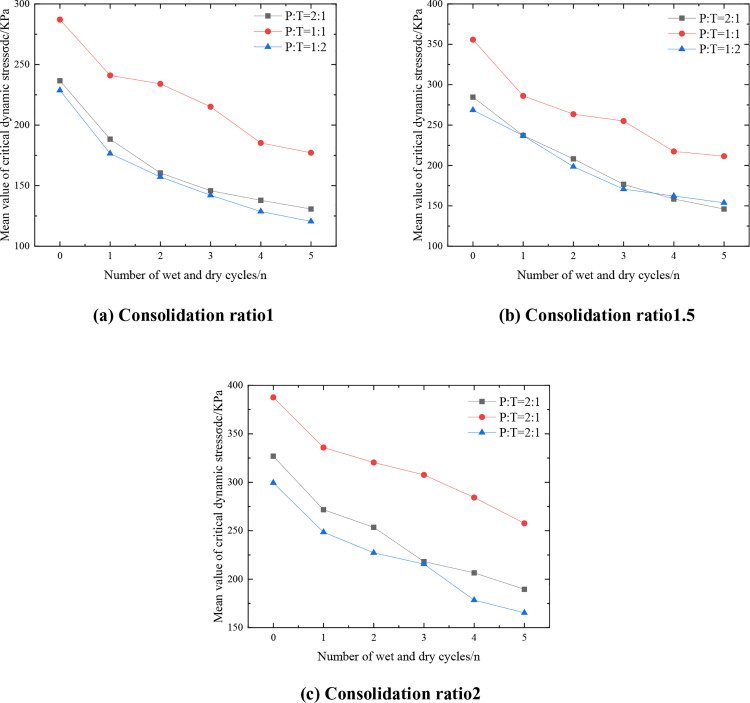
Variation curve of critical dynamic stress representative value of mixture with different mix ratio.

Fig 8 shows the variation curves of the representative values of critical dynamic stress and the number of wet and dry cycles of the mixes with different phosphogypsum dosage under 6% cement. From Fig 8, it can be seen that under the same number of wet and dry cycles, with the increase of phosphogypsum dosage, the critical dynamic stress of the mix first increases and then decreases, the critical dynamic stress of the mix is the largest when P:T = 1:1.

### 3.3. Critical dynamic stress sensitivity analysis of mixes

In order to investigate the degree of influence of the three factors of perimeter pressure, consolidation ratio and the number of wet and dry cycles on the critical dynamic stress of the mixture, according to the test data of the critical dynamic stress of C:P:T = 6:47:47, the orthogonal table of L9 (3^3^) was selected for orthogonal analysis, and the levels of the factors and the orthogonal table are shown in Table 13 and Table 14.

**Table 13:** Levels of various factors in orthogonal test.

Level	Pressurization σ_3_/kPa	Consolidation ratio	Number of dry and wet cycles *N*/times
1	40	1	0
2	80	1.5	3
3	120	2	5

**Table 14:** Orthogonal test table.

Test number	Pressurization	Consolidation ratio	Number of wet and dry cycles	Critical dynamic stress
1	1	1	1	218.22
2	1	2	2	183.75
3	1	3	3	192.51
4	2	1	2	215.12
5	2	2	3	211.51
6	2	3	1	419.93
7	3	1	3	258.18
8	3	2	2	255.00
9	3	3	1	451.69

SPSS software was used to process the experimental data and the orthogonal test results were obtained as shown in Table 15

**Table 15:** Orthogonal test results of mixture.

Independent variable	Degrees of freedom	Mean square	F	Significance	R^2^
pressurization	2	11929.46	9.99	0.091	0.88
consolidation ratio	2	5327.40	4.46	0.183	
Number of wet and dry cycles	2	1194.23	5.468	0.12	

From the F-values of the factors in the orthogonal test results, it can be seen that the enclosing pressure has the greatest influence on the critical dynamic stress of the mix, followed by the number of wet and dry cycles and the consolidation ratio, respectively.

### 3.4. Mechanistic analysis of dynamic strength of mixes

**Fig 9:**
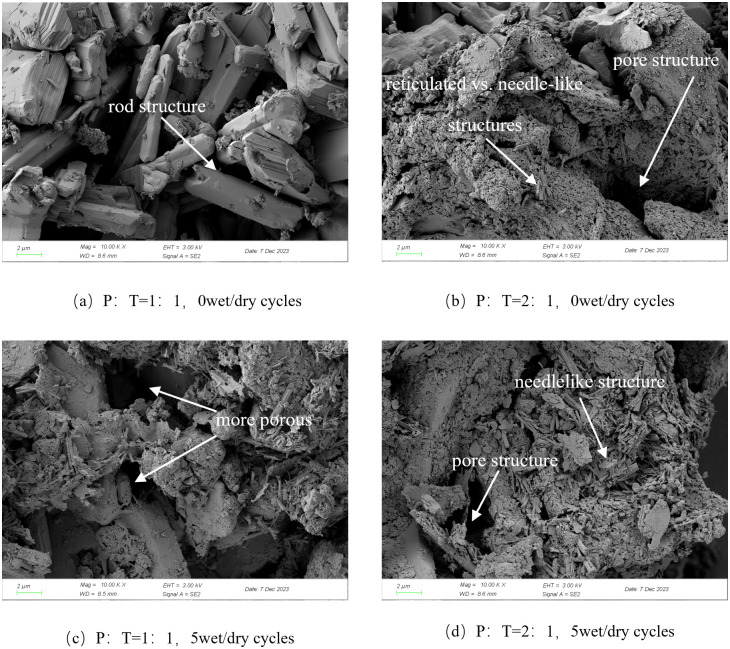
Scanning electron microscope diagram of optimally proportioned and over-portioned mix.

**Fig 10:**
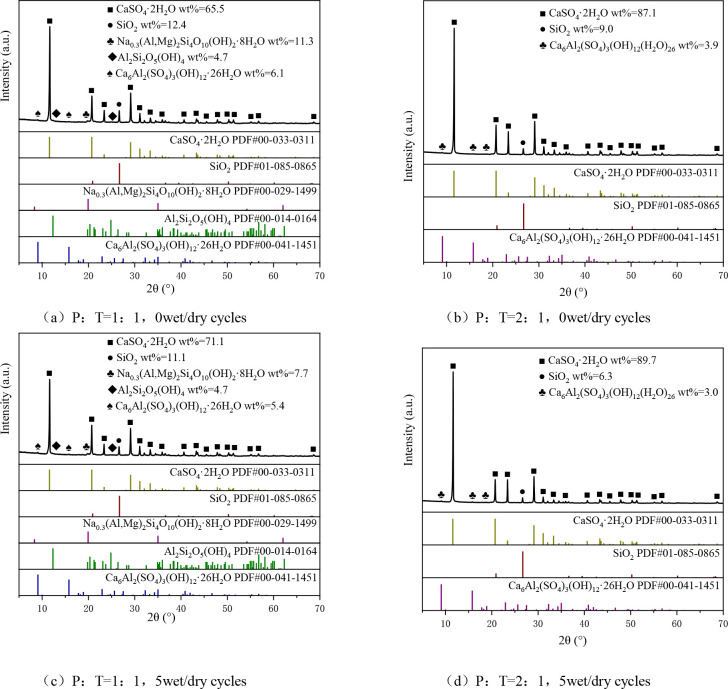
Physical phase identification diagram of optimally proportioned and over-portioned mix.

The formation of cement-phosphogypsum-red clay dynamic strength and the micro-mechanism of the change is a complex process involving the combined effect of various factors such as microstructural properties and chemical reactions, Fig 9 and 10 show the results of scanning electron microscope (SEM) and physical analysis of the mixes, respectively, which are analyzed in the following for mixes with different phosphogypsum mixing amounts and different numbers of dry and wet cycles.

#### 3.4.1. Effect of phosphogypsum dosing.

When P:T = 1:1, the particle size of the mix without dry and wet cycle is mostly distributed in 4 ~ 20μm, with a large number of rods intertwined to form a stable structure with a certain strength, which is mainly attributed to the following reasons: (1) The hydration reaction of cement in the mix generates hydrated calcium silicate gel (CSH), which has strong cementing ability and can coalesce the fine particles in the mix into clusters; (2) Phosphogypsum contains a large amount of calcium sulfate dihydrate (CSD), and SO₄^2^- released by dissolution of CSD is combined with C₃A in cement during the hydration process. in the hydration process, firstly generate monosulfur-type calcium sulfoaluminate (AFm), and then further transformed into calcium alumina (Aft) crystals in the continuous reaction. Under the microscopic view, Aft mainly shows columnar shape, which is intertwined with the hydrated calcium silicate to enhance the occlusion force of the material, thus constituting a stable system with a certain degree of strength, and increasing the dynamic strength of the mix, the specific reaction process as in Equation (4) ~ (6) [37]; ((3) The mixture contains Ca2 + , Fe3+ and other metal cations, which have a strong adsorption capacity for the electrostatically charged soil particles [38], and thus coalesce into a bond with higher strength.


$$\begin{array}{c} AlO_{2}^{-}+2OH+2H_{2}O={2\left[Al(OH{)}_{6}\right]}^{3-} \end{array}$$ (4)

$$\begin{array}{c} {2\left[Al(OH{)}_{6}\right]}^{3-}+{6Ca}^{2+}+24H_{2}O={\left\{{Ca}_{6}{\left[Al(OH{)}_{6}\right]}_{2}24H_{2}O\right\}}^{6+} \end{array}$$ (5)

$$\begin{array}{c} {\left\{{Ca}_{6}{\left[Al(OH{)}_{6}\right]}_{2}24H_{2}O\right\}}^{6+}+3SO_{4}^{2-}+2H_{2}O=\{{Ca}_{6}{\left[Al(OH{)}_{6}\right]}_{2}24H_{2}O\}[3SO_{4}^{2-}2H_{2}Ononumber \end{array}$$ (6)

When P:T = 2:1, there are a lot of flocculent material and needle-like material on the surface of the mixture, most of its particle size is less than 1 μm, and the structure is loose, indicating that with the increase of phosphogypsum doping, the particle size of the mixture has a tendency to decrease. From Fig 9: the relative content of calcium alumina in the mixture is 6.1% when P:T = 1:1, and 3.9% when P:T = 2:1, with the increase of phosphogypsum dosage, the relative content of Aft in the mixture does not increase but decreases, which is mainly due to the fact that the high dosage of phosphogypsum contains more SO_4_^2-^, making the mixture more acidic, and part of the Aft dissolved to produce Calcium carbonate and alumina, etc. [39], the microstructure shows flocculent and needle-like structure, the structural strength is reduced, and the critical dynamic stress of the mixture decreases. The presence of a large number of CSD crystals in the mix indicates that some of the phosphogypsum that was not involved in the reaction is filling in the pores of the soil as fine aggregate. However, phosphogypsum is less hard and without enough hydration products to encapsulate it will become a weak part of the mix and reduce the strength of the mix.

#### 3.4.2. Effects of wet and dry cycles.

Under the same ratio, a large number of needle stick material in the mixture without wet and dry cycle cross-lap to form a stable structure, but there is part of the pore, 5 wet and dry cycle after the original structure has been damaged to a certain extent, part of the needle stick material dissolved to form a loose mesh structure attached to the surface of the pore, the structural stability of the reduced. As can be seen from Fig 9, the montmorillonite content in the mixture decreased after the wet and dry cycles. Montmorillonite is a substance with strong hydrophilic ability. When the specimen is humidified, montmorillonite absorbs water and expands, and when it is dried, it loses water and shrinks, forming large pores inside the mix, increasing the porosity of the mix, part of the particles flow out of the specimen with the water, reducing the dynamic strength of the mix. 5 times of the wet and dry cycle the relative content of Aft in the mix decreased, which is one of the reasons for the reduction of the dynamic strength of the mix after the wet and dry cycle.

## 4. Reach a verdict

(1) Under the action of wet and dry cycles, the cumulative deformation of the mixture is positively correlated with the dynamic stress, the number of vibrations, the critical dynamic stress of the mixture increases with the increase of the peripheral pressure and consolidation ratio, and decreases with the increase of the number of wet and dry cycles, and the first wet and dry cycles of the mixture’s critical dynamic stress is the largest degree of attenuation
(2) The cumulative deformation curves of cemented phosphogypsum stabilized soil under dry and wet cycling are classified into three types: stabilized, destructive and critical, which can be fitted by, $\varepsilon_{p}=\frac{\alpha *N^{\beta }}{1+\gamma *N^{\beta }}$, $\varepsilon_{p}=AN^{B}$, respectively, with the first two fitted better and the critical type fitted poorly;
(3) Perimeter pressure has the greatest effect on the critical dynamic stress of cemented phosphogypsum stabilized soil, followed by the number of wet and dry cycles, and consolidation ratio, respectively;
(4) Cement hydration generates hydrated calcium silicate, electrostatic adsorption between charged particles to form clusters, cement and phosphogypsum reaction generates calcium alumina, a variety of agglomerates intertwined to form a stable structure, thus improving the dynamic strength of cement phosphogypsum stabilized soil;
(5) The mixture has good dynamic strength when the mass ratio of phosphogypsum to red clay is 1:1. When the ratio of phosphogypsum to red clay is more than 1:1, it will lead to the dissolution of part of calcite and reduce the occlusion force between the agglomerates, and at the same time, the dry and wet cycling effect will increase the porosity within the mixture and reduce the relative content of calcite, which reduces the mixture’s ability to resist the damage of dynamic loading.
(6) Since phosphogypsum contains soluble phosphorus, fluoride, heavy metals and other impurities, it slows down the hydration rate of the mix, increases the pore space, loosens the structure, and reduces the strength, leading to a decrease in durability. Secondly, different phosphogypsum sources have different impurity contents, and phosphogypsum leaching toxicity may affect the environment (groundwater and radioactivity), and it is recommended to strengthen the research on phosphogypsum leaching toxicity.

## Supporting information

S1 Data Minimum data set. (DOCX)
