# Supplementary material for: Fatigue characteristics and mechanism of phosphogypsum stabilised red clay under dry and wet cycles
Source: PLoS One. 2025 Sep 5;20(9):e0331317. doi: 10.1371/journal.pone.0331317 (PMC12413006; doi:10.1371/journal.pone.0331317)
Supplement: S1 Data — (DOCX) [file pone.0331317.s001.docx]

**Minimum data set**

Tab. 1 Basic physical indexes of red clay

| Natural density /g‧cm^-3^  1.765 | Natural moisture content /% | Unevenness coefficient | | Curvature factor | | Liquid limit /% |
| --- | --- | --- | --- | --- | --- | --- |
|  | 49.49 | 8.60 | | 1.40 | | 80.40 |
| Plastic limit /% | Plasticity index | | Optimum moisture content /% | | Maximum dry density /g‧cm^-3^ | |
| 49.60 | 30.80 | | 23.74 | | 1.452 | |

Tab. 2 Basic Parameters of Phosphogypsum and Phosphogypsum chemical composition

| Basic Parameters of Phosphogypsum | specific surface area / m^2^‧kg^-1^ | 102 |
| --- | --- | --- |
|  | heat loss /% | 18.43 |
|  | moisture content /% | 5.3 |
|  | densities /g‧cm^-3^ | 2.38 |
|  | fineness /% | 44.3 |
| Phosphogypsum chemical composition mass fraction（%） | SO_3_ | 49.070 |
|  | CaO | 40.070 |
|  | SiO_2_ | 5.780 |
|  | P_2_O_5_ | 1.350 |
|  | Na_2_O | 0.587 |
|  | Al_2_O_3_ | 0.435 |
|  | else | 2.708 |

Tab. 3 Test Results of Heavy Metals and Radioactivity in Phosphogypsum

| sports event | | limit value | Test results | reach a verdict |
| --- | --- | --- | --- | --- |
| heavy metal element | Cu/mg‧L^-1^ | ≤100 | 0.157 | eligible |
|  | Zn/mg‧L^-1^ | ≤100 | 0.051 | eligible |
|  | Cd/mg‧L^-1^ | ≤1 | 0 | eligible |
|  | Pb/mg‧L^-1^ | ≤5 | 0 | eligible |
|  | Cr/mg‧L^-1^ | ≤15 | 0 | eligible |
|  | As/mg‧L^-1^ | ≤5 | 0.0356 | eligible |
|  | Hg/mg‧L^-1^ | ≤0.1 | 0.0005 | eligible |
| radiant | Ra-226/Bq‧kg^-1^ | — | 53.94 | — |
|  | Th-232/Bq‧kg^-1^ | — | 42.13 | — |
|  | K-40/Bq‧kg^-1^ | — | 52.95 | — |
|  | internal irradiance index (IRI) | ≤1.0 | 0.3 | eligible |
|  | external irradiance index (EI) | ≤1.0 | 0.3 | eligible |

Tab. 4 Basic parameters of cement

| heat loss /% | SO3 /% | MgO/% | specific surface area /m2/kg | Incipient condensation time /min | time of final coagulation /min |
| --- | --- | --- | --- | --- | --- |
| 4.14 | 2.20 | 1.98 | 348 | 166 | 221 |
| stability | chloride ion /% | Gypsum Admixture /% | Grinding aids/% | 3-Day Flexural Strength /MPa | 3-day compressive strength /MPa |
| eligible | 0.018 | 5.00 | 0.1 | 5.9 | 29.2 |

Tab. 5 Optimum moisture content and maximum dry density of mixes with different proportions

| P：T | 4% C | | 6% C | | 8% C | |
| --- | --- | --- | --- | --- | --- | --- |
|  | Optimum moisture content /% | Maximum dry density /g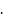cm^-3^ | Optimum moisture content /% | Maximum dry density /g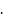cm^-3^ | Optimum moisture content /% | Maximum dry density /g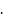cm^-3^ |
| 2：1 | 20.30 | 1.53 | 19.40 | 1.52 | 18.90 | 1.56 |
| 1：1 | 21.30 | 1.56 | 21.80 | 1.56 | 20.80 | 1.57 |
| 1：2 | 22.50 | 1.54 | 23.10 | 1.55 | 21.80 | 1.56 |
| 1：3 | 23.46 | 1.52 | 24.10 | 1.54 | 22.90 | 1.54 |
| 1：4 | 24.36 | 1.45 | 25.60 | 1.51 | 24.50 | 1.50 |
| 1：5 | 26.00 | 1.46 | 26.70 | 1.50 | 25.70 | 1.50 |

Tab. 6 Dry and wet cycle moisture content

| P：T | 4% C | | | 6% C | | | 8% C | | |
| --- | --- | --- | --- | --- | --- | --- | --- | --- | --- |
|  | Optimum moisture content /% | Humidified moisture content /% | Dry moisture content /% | Optimum moisture content /% | Humidified moisture content /% | Dry moisture content /% | Optimum moisture content /% | Humidified moisture content /% | Dry moisture content /% |
| 2：1 | 20.30 | 25.30 | 15.30 | 19.40 | 24.40 | 14.40 | 18.90 | 23.90 | 13.90 |
| 1：1 | 21.30 | 26.30 | 16.30 | 21.80 | 26.80 | 16.80 | 20.80 | 25.80 | 15.80 |
| 1：2 | 22.50 | 27.50 | 17.50 | 23.10 | 28.10 | 18.10 | 21.80 | 26.80 | 16.80 |
| 1：3 | 23.46 | 28.46 | 18.46 | 24.10 | 29.10 | 19.10 | 22.90 | 27.90 | 17.90 |
| 1：4 | 24.36 | 29.36 | 19.36 | 25.60 | 30.60 | 20.60 | 24.50 | 29.50 | 19.50 |
| 1：5 | 26.00 | 31.00 | 21.00 | 26.70 | 31.70 | 21.70 | 25.70 | 30.70 | 20.70 |

Tab. 7 Dynamic triaxial fatigue test programme

| frequency /（Hz） | pressurization（kPa） | consolidation ratio | Number of cycles*N*/次 |
| --- | --- | --- | --- |
| 5 | 40 | 1 | 0、1、2、3、4、5 |
|  |  | 1.5 | 0、1、2、3、4、5 |
|  |  | 2 | 0、1、2、3、4、5 |
| 5 | 80 | 1 | 0、1、2、3、4、5 |
|  |  | 1.5 | 0、1、2、3、4、5 |
|  |  | 2 | 0、1、2、3、4、5 |
| 5 | 120 | 1 | 0、1、2、3、4、5 |
|  |  | 1.5 | 0、1、2、3、4、5 |
|  |  | 2 | 0、1、2、3、4、5 |

Tab. 8 Dynamic stress amplitude values for the mix ratio 6:47:47

| consolidation ratio | pressurization /kPa | Dry and wet cycle  Number of cycles/times | Initial dynamic stress  Amplitude /kPa | Dynamic Stress  amplification /kPa |
| --- | --- | --- | --- | --- |
| 1 | 40 | 0~5 | 160,140,130,120,100,80 | 20 |
|  | 80 |  | 200,180,160,140,130,120 | 20 |
|  | 120 |  | 220,200,180,160,150,140 | 20 |
| 1.5 | 40 | 0~5 | 220,200,160,140,130,120 | 20 |
|  | 80 |  | 260,220,180,160,150,140 | 20 |
|  | 120 |  | 280,230,200,180,160,150 | 20 |
| 2 | 40 | 0~5 | 240,220,180,160,150,140 | 20 |
|  | 80 |  | 280,240,200,180,160,150 | 20 |
|  | 120 |  | 300,280,260,240,220,180 | 20 |

Tab. 9 Table of SEM and XRD test protocols

| proportion /P：T | Cement Admixture /% | moisture content /% | | compaction /% | Number of wet and dry cycles |
| --- | --- | --- | --- | --- | --- |
| 2：1  1：1 | 6 | 21.80（optimal）  23.10（optimal） | | 96 | 0、5  0、5 |
|  | | |  | | |
| (a) 0 wet/dry cycles | | | (b) 3wet/dry cycles | | |
|  | | | | | |
| (b) 5 wet/dry cycles | | | | | |

Fig. 4 Cumulative deformation ε_p_~N curves of mixes with different number of wet and dry cycles

Raw data for Figure 4

| 0 wet/dry cycles | | | | | | | | |
| --- | --- | --- | --- | --- | --- | --- | --- | --- |
| Number of vibrations *N*/times | Cumulative deformationε_p_(%) | | | | | | | |
| 1 | 0.6875 | 0.972 | 1.04 | 1.16 | 1.37 | 1.38 | 1.5 | 2 |
| 10 | 0.7056 | 0.9734 | 1.15 | 1.28 | 1.56 | 1.9 | 2.3 | 3.2 |
| 100 | 0.7243 | 1.051 | 1.23 | 1.54 | 2.05 | 2.54 | 3.5 | 4.5 |
| 300 | 0.772 | 1.198 | 1.36 | 1.66 | 2.36 | 2.913 | 4.1 | 5.5 |
| 500 | 0.811 | 1.2424 | 1.44 | 1.87 | 2.51 | 3.27 | 4.5 | 6 |
| 800 | 0.83 | 1.287 | 1.62 | 2.082 | 2.76 | 3.71 | 5.1 | 6.6 |
| 1000 | 0.87 | 1.363 | 1.754 | 2.17 | 3.03 | 4.08 | 5.37 | 6.97 |
| 2000 | 0.93 | 1.53 | 2.05 | 2.48 | 3.67 | 5.14 | 6.43 | 8.53 |
| 3000 | 1.02 | 1.66 | 2.35 | 2.75 | 4.33 | 5.86 | 7.59 | 9.84 |
| 4000 | 1.09 | 1.751 | 2.51 | 2.85 | 4.84 | 6.35 | 9.08 | 12.01 |
| 5000 | 1.16 | 1.94 | 2.64 | 3.02 | 6.53 | 7.08 | 9.51 | -- |
| 6000 | 1.24 | 2.03 | 2.72 | 3.26 | 6.32 | 7.62 | 10.06 | -- |
| 7000 | 1.38 | 2.08 | 2.85 | 3.42 | 6.75 | 8.04 | 10.5 | -- |
| 8000 | 1.41 | 2.18 | 2.94 | 3.56 | 6.19 | 8.57 | -- | -- |
| 9000 | 1.53 | 2.25 | 3.03 | 3.64 | 6.89 | 8.32 | -- | -- |

| 3 wet/dry cycles | | | | | | | | |
| --- | --- | --- | --- | --- | --- | --- | --- | --- |
| Number of vibrations *N*/times | Cumulative deformationε_p_(%) | | | | | | | |
| 1 | 0.6875 | 0.972 | 1.04 | 1.16 | 1.37 | 1.38 | 1.8 | 2 |
| 10 | 0.7056 | 0.9734 | 1.15 | 1.28 | 1.56 | 1.9 | 2.3 | 2.8 |
| 100 | 0.7243 | 1.051 | 1.23 | 1.74 | 2.05 | 2.54 | 3 | 3.5 |
| 300 | 0.772 | 1.198 | 1.36 | 1.96 | 2.36 | 2.913 | 3.7 | 4.1 |
| 500 | 0.811 | 1.2424 | 1.44 | 2.087 | 2.51 | 3.27 | 4 | 4.6 |
| 800 | 0.83 | 1.287 | 1.62 | 2.382 | 2.86 | 3.51 | 4.5 | 5.3 |
| 1000 | 0.87 | 1.363 | 1.754 | 2.57 | 3.03 | 3.68 | 4.87 | 5.57 |
| 2000 | 0.93 | 1.53 | 2.05 | 3.08 | 3.67 | 4.84 | 6.03 | 7.57 |
| 3000 | 1.02 | 1.66 | 2.35 | 3.25 | 4.33 | 5.56 | 7.59 | 9.29 |
| 4000 | 1.09 | 1.751 | 2.51 | 3.55 | 4.84 | 6.35 | 8.08 | 10.16 |
| 5000 | 1.16 | 1.94 | 2.64 | 3.802 | 5.43 | 6.98 | 9.06 | 10.87 |
| 6000 | 1.24 | 2.03 | 2.72 | 4.06 | 5.12 | 6.62 | 9.36 | -- |
| 7000 | 1.38 | 2.08 | 2.85 | 4.22 | 5.45 | 7.04 | 9.96 | -- |
| 8000 | 1.41 | 2.18 | 2.94 | 4.36 | 4.99 | 6.57 | -- | -- |
| 9000 | 1.53 | 2.25 | 3.03 | 4.64 | 5.89 | 7.32 | -- | -- |

| 5 wet/dry cycles | | | | | | | | |
| --- | --- | --- | --- | --- | --- | --- | --- | --- |
| Number of vibrations *N*/times | Cumulative deformationε_p_(%) | | | | | | | |
| 1 | 0.6875 | 0.972 | 1.04 | 1.16 | 1.37 | 1.38 | 1.5 | 2 |
| 10 | 0.7056 | 0.9734 | 1.15 | 1.28 | 1.56 | 1.9 | 2 | 2.8 |
| 100 | 0.7243 | 1.051 | 1.23 | 1.54 | 2.05 | 2.54 | 2.7 | 3.5 |
| 300 | 0.772 | 1.198 | 1.36 | 1.66 | 2.36 | 2.913 | 3.3 | 4.1 |
| 500 | 0.811 | 1.2424 | 1.44 | 1.87 | 2.51 | 3.27 | 3.8 | 4.6 |
| 800 | 0.83 | 1.287 | 1.62 | 2.082 | 2.96 | 3.51 | 4.5 | 5.3 |
| 1000 | 0.87 | 1.363 | 1.754 | 2.17 | 3.23 | 3.68 | 4.87 | 6.27 |
| 2000 | 0.93 | 1.53 | 2.05 | 2.48 | 3.67 | 4.44 | 5.43 | 7.39 |
| 3000 | 1.02 | 1.66 | 2.35 | 2.75 | 4.33 | 4.86 | 6.08 | 7.88 |
| 4000 | 1.09 | 1.751 | 2.51 | 2.85 | 4.84 | 5.35 | 6.51 | 8.31 |
| 5000 | 1.16 | 1.94 | 2.64 | 3.02 | 5.43 | 5.98 | 7.06 | 8.86 |
| 6000 | 1.24 | 2.03 | 2.72 | 3.26 | 5.12 | 5.62 | 7.76 | 10 |
| 7000 | 1.38 | 2.08 | 2.85 | 3.42 | 5.45 | 6.04 | -- | -- |
| 8000 | 1.41 | 2.18 | 2.94 | 3.56 | 4.99 | 5.57 | -- | -- |
| 9000 | 1.53 | 2.25 | 3.03 | 3.64 | 5.89 | 6.32 | -- | -- |

Tab. 10 Cumulative deformation curve fitting parameters for 0 wet and dry cycles of mixes

| Curve Type | Dynamic Stress Amplitude Setting /kPa | Dynamic stress amplitude measured value /kPa | α,a,A | β,b,B | γ | R^2^ |
| --- | --- | --- | --- | --- | --- | --- |
| stabilized | 260 | 263.01 | 0.03169 | 0.43361 | 0.01480 | 0.91094 |
| stabilized | 280 | 285.15 | 0.00701 | 0.91446 | 0.00563 | 0.96323 |
| stabilized | 300 | 301.41 | 0.14352 | 0.43149 | 0.03352 | 0.96281 |
| stabilized | 320 | 323.84 | 0.09815 | 0.59101 | 0.02350 | 0.96453 |
| critical | 340 | 342.54 | 1.94364 | 0.00044 | --- | 0.84728 |
| critical | 360 | 368.97 | 2.41525 | 0.00067 | --- | 0.83705 |
| destructive | 380 | 380.71 | 0.35797 | 0.43580 | --- | 0.98230 |
| destructive | 400 | 401.3 | 0.47937 | 0.46391 | --- | 0.95675 |

Tab. 11 Cumulative deformation curve fitting parameters for 3 wet and dry cycles of mixes

| Curve Type | Dynamic Stress Amplitude Setting /kPa | Dynamic stress amplitude measured value /kPa | α,a,A | β,b,B | γ | R^2^ |
| --- | --- | --- | --- | --- | --- | --- |
| stabilized | 160 | 161.33 | 0.00370 | 0.73071 | 0.00473 | 0.94258 |
| stabilized | 180 | 185.42 | 0.00522 | 1.00106 | 0.00360 | 0.90689 |
| stabilized | 200 | 201.34 | 0.09470 | 0.46181 | 0.02885 | 0.95492 |
| stabilized | 220 | 223.46 | 0.25391 | 0.35622 | 0.02550 | 0.98007 |
| critical | 240 | 242.72 | 1.87610 | 0.00045 | --- | 0.85771 |
| critical | 260 | 268.75 | 2.27895 | 0.00066 | --- | 0.86605 |
| destructive | 280 | 284.12 | 0.40704 | 0.39665 | --- | 0.99039 |
| destructive | 300 | 297.25 | 0.44113 | 0.43261 | --- | 0.98805 |

Tab. 12 Cumulative deformation curve fitting parameters for 5 wet and dry cycles of mixes

| Curve Type | Dynamic Stress Amplitude Setting /kPa | Dynamic stress amplitude measured value /kPa | α,a,A | | β,b,B | γ | R^2^ |
| --- | --- | --- | --- | --- | --- | --- | --- |
| stabilized | 140 | 143.26 | 0.07365 | | 0.23744 | 0.11066 | 0.84888 |
| stabilized | 160 | 165.61 | 0.02409 | | 0.79659 | 0.01924 | 0.89569 |
| stabilized | 180 | 181.06 | 0.08023 | | 0.70132 | 0.03574 | 0.92444 |
| stabilized | 190 | 193.84 | 0.09464 | | 0.55516 | 0.02136 | 0.96382 |
| critical | 200 | 201.30 | 2.25006 | | 0.00040 | --- | 0.73010 |
| critical | 220 | 222.27 | 2.41597 | | 0.00061 | --- | 0.88115 |
| destructive | 240 | 240.54 | 0.81885 | | 0.28199 | --- | 0.99501 |
| destructive | 260 | 260.78 | 1.09132 | | 0.27924 | --- | 0.99662 |
|  | | | |  | | | |
| (a) Consolidation ratio1 | | | | (b) Consolidation ratio1.5 | | | |
|  | | | | | | | |
| (c) Consolidation ratio2 | | | | | | | |

Fig. 5 Curve of critical dynamic stress versus the number of wet and dry cycles

Raw data for Figure 5

| Consolidation ratio1 | | | |
| --- | --- | --- | --- |
| number of wet and dry cycles | Mean value dynamic stressσ_dc_/kPa | | |
| 0 | 218.9924 | 287.118 | 338.176 |
| 1 | 183 | 241 | 309.84 |
| 2 | 168 | 234 | 301 |
| 3 | 158.9924 | 215.118 | 290.176 |
| 4 | 128 | 185.21 | 268.6 |
| 5 | 118.9924 | 177.118 | 258.176 |

| Consolidation ratio1.5 | | | | | |
| --- | --- | --- | --- | --- | --- |
| number of wet and dry cycles | Mean value dynamic stressσ_dc_/kPa | | | | |
| 0 | 275.7487 | 355.75 | | 419.9323 | |
| 1 | 215 | 286.04 | | 347.24 | |
| 2 | 192.43 | 263.47 | | 340.41 | |
| 3 | 183.7487 | | 255 | | 329.9323 |
| 4 | 171 | | 217.3 | | 305 |
| 5 | 165.7487 | | 211.5 | | 299.9323 |

| Consolidation ratio2 | | | |
| --- | --- | --- | --- |
| number of wet and dry cycles | Mean value dynamic stressσ_dc_/kPa | | |
| 0 | 312.505 | 387.6306 | 451.6886 |
| 1 | 266 | 335.87 | 410.7 |
| 2 | 254.61 | 320.42 | 390.64 |
| 3 | 232.505 | 307.6306 | 371.6886 |
| 4 | 215 | 284.27 | 348.77 |
| 5 | 192.505 | 257.6306 | 331.6886 |

|  |  |
| --- | --- |
| (a) 0 wet/dry cycles | (b) 3 wet/dry cycles |
|  | |
| (c) 5 wet/dry cycles | |

Fig. 6 Variation curve of mean critical dynamic stress-consolidation ratio of mixture

Raw data for Figure 6

| 0 wet/dry cycles | | | |
| --- | --- | --- | --- |
| peripheralσ_3_/kPa |  | Mean value dynamic stressσ_dc_/kPa |  |
| 40 | 218.9924 | 275.7487 | 312.505 |
| 80 | 287.118 | 342.54 | 387.6306 |
| 120 | 338.176 | 419.9323 | 451.6886 |

| 3 wet/dry cycles | | | |
| --- | --- | --- | --- |
| peripheralσ_3_/kPa |  | Mean value dynamic stressσ_dck_KPa |  |
| 40 | 158.9924 | 183.7487 | 232.505 |
| 80 | 215.118 | 255 | 307.6306 |
| 120 | 290.176 | 329.9323 | 371.6886 |

| 5 wet/dry cycles | | | |
| --- | --- | --- | --- |
| peripheralσ_3_/kPa |  | Mean value dynamic stressσ_dc_/kPa |  |
| 40 | 118.9924 | 165.7487 | 192.505 |
| 80 | 177.118 | 211.5 | 257.6306 |
| 120 | 258.176 | 299.9323 | 331.6886 |

|  |  |
| --- | --- |
| (a) 0 wet/dry cycles | (b) 3 wet/dry cycles |
|  | |
| (c) 5 wet/dry cycles | |

Fig. 7 Variation curve of mean critical dynamic stress-consolidation ratio of mixture

Raw data for Figure 7

| 0 wet/dry cycles | | | |
| --- | --- | --- | --- |
| Consolidation ratio | Mean value dynamic stress σ_dc_/kPa | | |
| 1 | 218.9924 | 287.118 | 338.176 |
| 1.5 | 275.748 | 342.54 | 419.9323 |
| 2 | 312.505 | 419.9323 | 451.6886 |

| 3 wet/dry cycles | | | |
| --- | --- | --- | --- |
| Consolidation ratio | Mean value dynamic stress σ_dc_/kPa | | |
| 1 | 158.9924 | 215.118 | 290.176 |
| 1.5 | 183.7487 | 255 | 329.9323 |
| 2 | 232.505 | 307.63 | 371.6886 |

| 5 wet/dry cycles | | | |
| --- | --- | --- | --- |
| Consolidation ratio | Mean value dynamic stress σ_dc_/kPa | | |
| 1 | 118.9924 | 177.118 | 258.176 |
| 1.5 | 165.7487 | 211.5 | 299.9323 |
| 2 | 192.505 | 257.6306 | 331.6886 |

|  |  |
| --- | --- |
| (a) Consolidation ratio1 | (b) Consolidation ratio1.5 |
|  | |
| (c) Consolidation ratio2 | |

Fig. 8 Variation curve of critical dynamic stress representative value of mixture with different mix ratio

Raw data for Figure 8

| Consolidation ratio1 | | | |
| --- | --- | --- | --- |
| Number of wet and cycles/n | Mean value dynamic stress σ_dc_/kPa | | |
| 0 | 236.56 | 287.118 | 228.64 |
| 1 | 188.32 | 241 | 176.45 |
| 2 | 160.45 | 234 | 157.21 |
| 3 | 145.78 | 215.118 | 142.01 |
| 4 | 137.84 | 185.21 | 128.63 |
| 5 | 130.65 | 177.118 | 120.45 |

| Consolidation ratio1.5 | | | |
| --- | --- | --- | --- |
| Number of wet and cycles/n | Mean value dynamic stress σ_dc_/kPa | | |
| 0 | 284.51 | 355.75 | 268.5 |
| 1 | 237.06 | 286.04 | 236.8 |
| 2 | 208.12 | 263.47 | 198.5 |
| 3 | 176.54 | 255 | 170.64 |
| 4 | 158.37 | 217.3 | 162.1 |
| 5 | 146 | 211.5 | 153.76 |

| Consolidation ratio2 | | | |
| --- | --- | --- | --- |
| Number of wet and cycles/n | Mean value dynamic stress σ_dc_/kPa | | |
| 0 | 326.9 | 387.6306 | 299.42 |
| 1 | 271.64 | 335.87 | 248.48 |
| 2 | 253.5 | 320.42 | 227.19 |
| 3 | 218.12 | 307.6306 | 215.6 |
| 4 | 206.54 | 284.27 | 178.37 |
| 5 | 189.55 | 257.6306 | 165.3 |

Tab. 13 Levels of various factors in orthogonal test

| level | pressurization 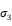/kPa | consolidation ratio | Number of dry and wet cycles N/times |
| --- | --- | --- | --- |
| 1 | 40 | 1 | 0 |
| 2 | 80 | 1.5 | 3 |
| 3 | 120 | 2 | 5 |

Tab. 14 Orthogonal test table

| test number | pressurization | consolidation ratio | Number of wet and dry cycles | Critical dynamic stress |
| --- | --- | --- | --- | --- |
| 1 | 1 | 1 | 1 | 218.22 |
| 2 | 1 | 2 | 2 | 183.75 |
| 3 | 1 | 3 | 3 | 192.51 |
| 4 | 2 | 1 | 2 | 215.12 |
| 5 | 2 | 2 | 3 | 211.51 |
| 6 | 2 | 3 | 1 | 419.93 |
| 7 | 3 | 1 | 3 | 258.18 |
| 8 | 3 | 2 | 2 | 255.00 |
| 9 | 3 | 3 | 1 | 451.69 |

Tab. 15 Orthogonal test results of mixture

| independent variable | degrees of freedom | mean square | F | significance | 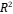 |
| --- | --- | --- | --- | --- | --- |
| pressurization | 2 | 11929.46 | 9.99 | 0.091 | 0.88 |
| consolidation ratio | 2 | 5327.40 | 4.46 | 0.183 |  |
| Number of wet and dry cycles | 2 | 1194.23 | 5.468 | 0.12 |  |
